# Three-Dimensional Convolutional Autoencoder Extracts Features of Structural Brain Images With a “Diagnostic Label-Free” Approach: Application to Schizophrenia Datasets

**DOI:** 10.3389/fnins.2021.652987

**Published:** 2021-07-07

**Authors:** Hiroyuki Yamaguchi, Yuki Hashimoto, Genichi Sugihara, Jun Miyata, Toshiya Murai, Hidehiko Takahashi, Manabu Honda, Akitoyo Hishimoto, Yuichi Yamashita

**Affiliations:** ^1^Department of Information Medicine, National Center of Neurology and Psychiatry, National Institute of Neuroscience, Tokyo, Japan; ^2^Department of Psychiatry, School of Medicine, Yokohama City University, Yokohama, Japan; ^3^Department of Psychiatry and Behavioral Sciences, Graduate School of Medical and Dental Sciences, Tokyo Medical and Dental University, Tokyo, Japan; ^4^Department of Psychiatry, Graduate School of Medicine, Kyoto University, Kyoto, Japan

**Keywords:** deep learning, machine learning, neuroimaging, schizophrenia, structural MRI, convolutional autoencoder, diagnostic label

## Abstract

There has been increasing interest in performing psychiatric brain imaging studies using deep learning. However, most studies in this field disregard three-dimensional (3D) spatial information and targeted disease discrimination, without considering the genetic and clinical heterogeneity of psychiatric disorders. The purpose of this study was to investigate the efficacy of a 3D convolutional autoencoder (3D-CAE) for extracting features related to psychiatric disorders without diagnostic labels. The network was trained using a Kyoto University dataset including 82 patients with schizophrenia (SZ) and 90 healthy subjects (HS) and was evaluated using Center for Biomedical Research Excellence (COBRE) datasets, including 71 SZ patients and 71 HS. We created 16 3D-CAE models with different channels and convolutions to explore the effective range of hyperparameters for psychiatric brain imaging. The number of blocks containing two convolutional layers and one pooling layer was set, ranging from 1 block to 4 blocks. The number of channels in the extraction layer varied from 1, 4, 16, and 32 channels. The proposed 3D-CAEs were successfully reproduced into 3D structural magnetic resonance imaging (MRI) scans with sufficiently low errors. In addition, the features extracted using 3D-CAE retained the relation to clinical information. We explored the appropriate hyperparameter range of 3D-CAE, and it was suggested that a model with 3 blocks may be related to extracting features for predicting the dose of medication and symptom severity in schizophrenia.

## Introduction

Deep learning (DL) has dramatically improved technology in speech recognition, image recognition, and many other fields (LeCun et al., 2015). Medical imaging can benefit greatly from recent progress in image classification and object detection using this cutting-edge technology (Esteva et al., 2019). In particular, as the global burden of psychiatric disorders increases (Olesen et al., 2012; Whiteford et al., 2013), psychiatric brain imaging studies using DL are anticipated to bring many benefits to society (Vieira et al., 2017). There are two major concerns about applying DL to psychiatric brain imaging: (1) treatment of the high dimensionality of data, and (2) the heterogeneity of psychiatric disorders (Feczko et al., 2019).

The dimensionality of raw magnetic resonance imaging (MRI) data is very high (often running into the millions), and large computer resources are required to analyze them. To reduce computational demands, in most neuroimaging studies, several feature extraction methods have been used. Region of interest (ROIs), one of the most popular feature extraction methods, has contributed to detecting various structural and functional abnormalities in the brains of patients with psychiatric disorders (Fornito et al., 2012; Fusar-Poli et al., 2012; Linden, 2012; Ratnanather et al., 2013). ROIs (often dozens or hundreds) are usually set based on neuroscience knowledge (Tzourio-Mazoyer et al., 2002). For example, average gray matter volumes or cortical thicknesses at specific ROIs are extracted as feature, and then the relationship between the feature and disease clinical information is analyzed (Desikan et al., 2006; Poldrack, 2007; Nelson et al., 2017). Even in the studies using DL, ROI-based features are often used as input (Vieira et al., 2017; Heinsfeld et al., 2018; Pinaya et al., 2019). In addition, many DL studies avoid using three-dimensional (3D) images directly, but instead, DL networks are trained using two-dimensional slices (Sarraf et al., 2017; Vieira et al., 2017; Aghdam et al., 2019). A limitation of these studies is that they ignore the 3D spatial information contained within the original MRI scans.

In recent years, with improvements in computer performance and refinement of computational techniques, studies have investigated how to treat 3D MRI scans as inputs to DL. For example, Wang et al. (2018) successfully discriminated Alzheimer’s dementia from healthy subjects using 3D MRI data as input to DL. Similar attempts have been made for discriminating psychiatric disorders, including schizophrenia (Qureshi et al., 2019) and developmental disorders (Wang et al., 2019). Although these studies demonstrated that DL could apply to the analysis of 3D MRI data, discrimination-based approaches may be challenging due to the heterogeneity of psychiatric disorders.

Heterogeneity is one of the main challenges that current psychiatric research faces (Feczko et al., 2019). The current symptom-based definitions of psychiatric disorders, standardized in the Diagnostic and Statistical Manual of the American Psychiatric Association (DSM) (American Psychiatric Association., 2013) and the International Classification of Diseases (ICD) (World Health Organization., 1992), have been highlighted as lacking predictive and clinical validity due to genetic and clinical heterogeneity (Owen, 2014). For example, in schizophrenia, a recent study found evidence for significant overlapping of the relatively common risk variants tagged in genome-wide association studies (GWAS) between several psychiatric disorders, and there may also be lower genetic correlation within disorders (Lee et al., 2014). In addition, even in patients given the same diagnosis of schizophrenia, the severity of symptoms, response to medication, and prognosis often vary widely among patients (van Os and Kapur, 2009; Owen et al., 2016). Therefore, in psychiatric disorders research, a simple competition for discrimination accuracy based on the current disorder categories may be insufficient to elucidate on pathophysiology, although most current studies using DL are attempting to discriminate disease in healthy subjects (Plis et al., 2014; Vieira et al., 2017; Gao et al., 2021; Quaak et al., 2021).

One possible alternative direction for using DL techniques in psychiatric neuroimaging studies may be diagnostic label-free feature extraction. In the current study, we focus on an autoencoder (AE) as a DL algorithm that allows feature extraction without labels (Hinton, 2006). AE is supervised learning in a deep neural network having an output layer with the same data as the input layer. Since the input is as supervision, no labels are needed, unlike in general supervised learning.

Indeed, there are some studies that have used AE-based feature extraction for psychiatric neuroimaging. For example, Pinaya et al. (2019) extracted features from structural MRI scans using AE, i.e., without using diagnostic labels. The authors successfully predicted the age and gender of participants, and discriminated patients with autism spectrum disorders (ASD) and schizophrenia from healthy subjects. However, these studies used ROI-based features such as cortical thickness and functional connectivity as inputs to the AE. As such, the use of 3D brain images for inputs to the AE remains challenging, with a few exceptions. For example, Martinez-Murcia et al. (2020) extracted features from 3D brain MRI data of patients with Alzheimer’s dementia using a 3D convolutional autoencoder (3D-CAE). They demonstrated that the extracted feature was useful for predicting age and Mini-Mental State Examination (MMSE) scores. This supports the efficacy of labeling free features based on 3D-CAE with MRI. However, particularly when investigating psychiatric disorders, the appropriate architecture of 3D-CAE has not been fully investigated.

The purpose of this study was to investigate an efficient 3D-CAE-based feature extraction for the neuroimaging of psychiatric disorders. More specifically, in the current study, we used datasets that included patients with schizophrenia, which has frequently been reported to be heterogeneous in previous neuroimaging studies (Sugihara et al., 2017). The key points of our study are: (1) to use 3D MRI data while preserving spatial information, and (2) diagnostic label-free feature extraction using 3D-CAE. For this purpose, we explored appropriate network structures of 3D-CAE by developing models with different network structures and comparing the predictive performance of clinical information by these extracted features.

## Materials and Methods

### Experimental Overview

**FIGURE 1:**
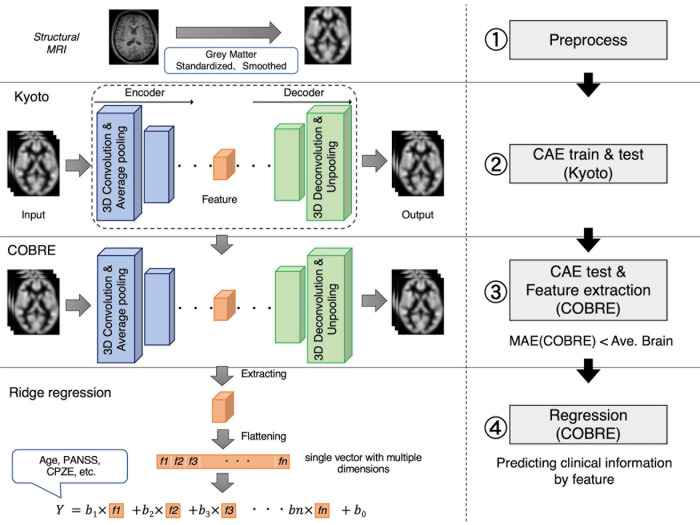
Experimental overview. 1. Preprocessing: The gray matter was extracted from the structural MRI and standardized and smoothed using SPM. 2. CAE training: A schematic diagram is shown. 3D images of the Kyoto dataset were input, feature was extracted, and the original image was reproduced. 3. Feature extraction: the model trained using the Kyoto dataset was adopted to the COBRE dataset without updating the weights. 4. Linear regression: Feature are extracted and flattened. Each extracted feature vectors were an explanatory variable, and demographic and clinical information were objective variables. Regression errors were evaluated to investigate whether the extracted features retain the information to predict demographic and clinical information. 3D, three-dimensional; CAE, convolutional autoencoder; COBRE, Center for Biomedical Research Excellence; CPZE, dose of antipsychotic medication; MRI, magnetic resonance imaging; SPM, Statistical Parametric Mapping.

Figure 1 illustrates an experimental overview of our study. We used two datasets, including participants diagnosed with schizophrenia as well as healthy subjects: a dataset collected at Kyoto University (Kyoto dataset) and a public dataset, The Center for Biomedical Research Excellence (COBRE^1^) dataset. (1) Gray matter was first extracted from the structural MRI data as preprocessing. (2) We then trained 3D-CAE to extract a latent feature representation from structural MRI using the Kyoto dataset. Sixteen 3D-CAEs with varying network structures were prepared for investigation of the optimal network depth and complexity. (3) Subsequently, the COBRE dataset was used to evaluate the applicability to another dataset. (4) Finally, we evaluated whether the extracted feature retained clinical information by linear regression of the clinical information using the COBRE dataset.

### Convolutional Autoencoder Training

An autoencoder is a kind of DL consisting of the encoder and the decoder. The encoder learns latent representations and reduces the dimension of the input. The decoder learns to reproduce the input as close as possible to the original using the latent representations. 3D-CAE extends this architecture by using convolutional layers that can extract features directly from 3D images (Guo et al., 2017; Nishio et al., 2017; Oh et al., 2019). The CAE has two main hyper parameters: the number of convolutional layers and the number of channels, which are the target of the current study.

The convolutional layers apply a filter to input to create feature maps that summarize the feature detected in the input. The feature maps are created for the number of channels. Since the convolutional layer generates feature maps while capturing the spatial information of the matrix, convolutional neural networks are beneficial to learning features of images. As the number of channels increases, the complexity of a model increases, but the number of dimensions of latent feature increase and requires a huge amount of computational power. Also, as the number of convolutions increases, the effective receptive field increases, thus allowing global and abstract feature to be extracted. The effective receptive field is a region of the original image that can potentially influence the activation of neurons (Le and Borji, 2017; Luo et al., 2017). If the effective receptive field is small, the feature will contain only local information of the brain, and if it is large, it will contain information on the whole brain.

**FIGURE 2:**
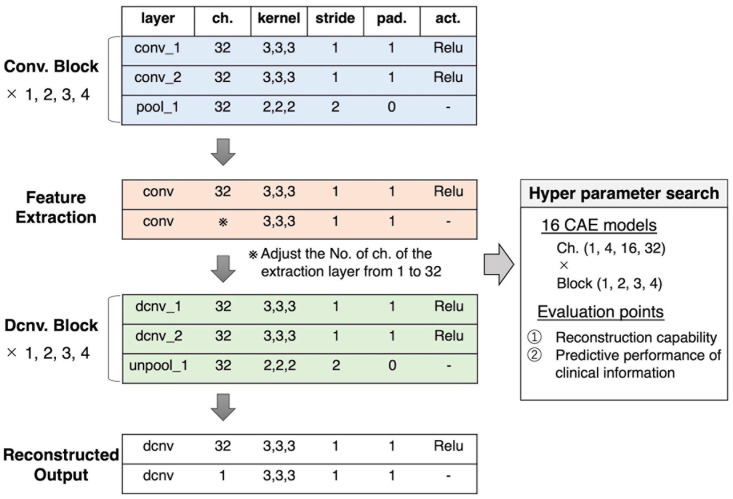
Our proposed 3D-CAE architecture. One convolution/deconvolution block was defined as repeating two convolution/deconvolution layers and one pooling/unpooling layer. The number of blocks was set from 1 to 4. The number of channels in the extraction layer was set from 1 to 32. Sixteen patterns of models with different numbers of blocks and channels were developed. In order to explore the effective number of channels and blocks, the reproduction capability and relation to clinical information were evaluated. act., activation function; 3D-CAE, three-dimensional convolutional autoencoder; ch., channel; Conv., convolution; Dcnv, deconvolution; pad., padding; pool, pooling; Relu, Rectified Linear Unit; unpool, unpooling.

In this study, these two hyperparameters were explored to investigate whether the total dimensions of the extracted feature and the size of the effective receptive field affected the relation of the feature to clinical information. As shown in Figure 2, the set of two convolution/deconvolution layers, and one pooling/unpooling layer was defined as a convolution/deconvolution “block.” In this experiment, the number of blocks was set, ranging from 1 block to 4 blocks. In 4 blocks, the effective receptive field is the whole brain; in 3 blocks, it is about 30% of the brain (multiple lobes), in 2 blocks, it is 5% of the brain (multiple regions), and in 1 block it is 0.1% of the brain (1 region). The number of channels in the extraction layer was varied with 1, 4, 16, and 32 channels, but the number of channels for other layers were fixed at 32. The number of channels was considered limited to 32 due to the limitation of the current experiment’s computational power. As a result, we created sixteen 3D-CAE models (4 block conditions × 4 channel conditions) to explore the effective range of hyperparameters for psychiatric brain imaging.

Other hyperparameters were fixed and common among models. The encoder was composed of convolution layers (a kernel size of 3 × 3 × 3 and a stride of 1) with rectified linear unit (ReLU) activations and average pooling layers (a kernel size of 2 × 2 × 2 and a stride of 2). The decoder was composed of convolution layers (a kernel size of 3 × 3 × 3 and a stride of 1) with ReLU activations and unpooling layers (a kernel size of 2 × 2 × 2 and a stride of 2). The loss function, consisting of the mean absolute error (MAE) between the input images and the reproduced images, was defined as follows:


(1) $$L\,o\,s\,s=\frac{1}{n}\,\sum \left|X_{i\,n\,p\,u\,t}-X_{r\,e\,c\,o\,n\,s\,t\,r\,u\,c\,t\,e\,d}\right|$$

As an optimizer, we used a gradient-based method with adaptative learning rates called Adam (Kingma and Ba, 2015) (alpha = 0.0001, beta1 = 0.9, beta2 = 0.999) using mini-batches with a size of eight samples. The training process was performed with a maximum of 50,000 training iterations. We conducted the experiments in Python 3.6^2^ using the Chainer v.5.4.0 library (Tokui et al., 2015).

We used a reference of training performances of 3D-CAEs, referred to as the “average brain,” with which the model was assumed to output the average intensities of the training dataset regardless of the inputs. The average brain is one of the most trivial solutions where the network outputs an image without learning any information about individual differences of the inputs. The average brain was used as a reference point to indicate that the model at least reproduced individual differences. The signal intensities of voxel *i* of the average brain was determined as follows:


(2) $$x_{a\,v\,e\,i=\frac{\sum_{s=0}^{n}x_{s,i}}{n}}$$

where *s* is a sample from the training dataset and *n* is the number of samples.

### Regression Analysis With Demographic and Clinical Information

Using trained 3D-CAE, latent feature vector could be extracted, and then the feature vector was flattened. The number of dimensions of that feature vector ranged from millions to hundreds, depending on the model. The relationship between the extracted feature and the clinical information was examined using regression analysis, based on the assumption that if the extracted feature is “informative,” it could help predict schizophrenia patients’ clinical information. Therefore, we confirmed this by comparing the prediction performance of 3D-CAE-based features and conventional ROI-based features. The linear regression analysis was performed with clinical and demographic information as the objective variables and the feature vectors as the explanatory variables (see the lower part of Figure 1). Demographic and clinical information included age, scores of positive and negative symptoms (PANSS), the dose of antipsychotic medications [chlorpromazine equivalent (CPZE)], Wechsler Adult Intelligence Scale (WAIS), duration of illness, age at onset, and diagnosis. For the regression analysis, in order to reduce the effects of correlated variables we adopted ridge regression, one of regularized linear regression methods. In the regression analysis, we executed a fivefold cross-validation process whereby the COBRE dataset was randomly divided into five groups of samples (folds), and then samples from fourfolds were used for training the regression model, and the other fold was used for the test of the regression model. The fivefold cross-validation was repeated ten times. The performance of the regression model was evaluated using the root mean square error (RMSE). The diagnosis was evaluated using accuracy.

Differences in the performances of regression models were evaluated using the two-way (number of channels × number of blocks) analysis of variance (ANOVA). Subsequently, Tukey’s multiple comparison test was performed for each group as a *post hoc* analysis. The level of significance was set to 0.05.

The 3D-CAE models were also compared with the ROI method. In the ROI method, using the automated anatomical labeling (AAL) template (Tzourio-Mazoyer et al., 2002), the GM was divided into 116 ROIs. The average intensities of each ROI were used as the ROI-based feature for regression analysis. The Student’s *t*-test was performed to compare the proposed 3D-CAE model with the ROI method. The level of significance was set to 0.05.

By calculating the gradient of the neural network at the input T1-weighted image for each subject, it is possible to visualize which regions of the input have higher weights. In this study, we attempted to visualize the regions that contribute to predicting clinical information by calculating the gradient of a composite function of feature extraction and clinical information regression functions. The calculation of a saliency map for input image *x*, *M(x)*, was defined as follows.


(3) M⁢(x)=∂⁡R⁢(S⁢(x))/∂⁡x

Where, *S()* was a feature extraction function based on the 3D-CAE, and *R()* was a function predicting clinical information using linear regression. To refine the visualization, the gradients’ calculation was repeated by adding Gaussian noise to the original image, similar to the technique used in SmoothGrad (Smilkov et al., 2017). The maps were then averaged by overall samples and divided by the standard deviation to obtain a *t*-value, and the values were finally converted to absolute values to yield a 3D saliency map.

### Kyoto Dataset Description

A total of 172 subjects were investigated in this study, including 82 patients with schizophrenia and 90 healthy subjects. Patients were recruited from hospitals in Kyoto, Japan, and diagnosed by psychiatrists using the Diagnostic and Statistical Manual of Mental Disorders, 4th edition (DSM-IV) (American Psychiatric Association., 1994) criteria for schizophrenia, confirmed with the patient edition of the Structured Clinical Interview for DSM-IV Axis I Disorders (SCID) (First et al., 1997). No patients had any comorbid DSM-IV Axis I disorder. The clinical symptoms of all patients were estimated using the Positive and Negative Syndrome Scale (PANSS) (Kay et al., 1987). Healthy subjects were screened with the non-patient edition of the SCID, confirming no history of psychiatric disorders. Exclusion criteria for all individuals included a history of head trauma, neurological illness, serious medical or surgical illness, or substance abuse. Note that participants were already diagnosed in order to expedite the data collection, but the diagnostic labels were not used to train the networks.

All participants were scanned with a 3.0-Tesla Siemens Trio scanner (Siemens Healthineers, Erlangen, Germany). The scanning parameters of the T1-weighted 3D magnetization-prepared rapid gradient-echo (3D-MPRAGE) sequences were as follows: echo time (TE) = 4.38 ms; repetition time (TR) = 2,000 ms; inversion time (TI) = 990 ms; field of view (FOV) = 225 mm × 240 mm; acquisition matrix size = 240 × 256 × 208; resolution = 0.9375 × 0.9375 × 1.0 mm^3^.

### COBRE Dataset Description

In this study, the COBRE dataset, which is a public dataset, was acquired as a dataset with different scanning sites and parameters to the Kyoto University dataset. All the subjects were diagnosed and screened with the SCID. The clinical symptoms of all patients were estimated using the PANSS. Exclusion criteria for individuals included a history of head trauma, neurological illness, serious medical or surgical illness, or substance abuse. We included a total of 142 subjects from this database in our study, including 71 patients with schizophrenia and 71 healthy subjects.

MRI data were acquired using a 3.0-Tesla Siemens Tim Trio scanner (Siemens Healthineers, Erlangen, Germany). The scanning parameters of the T1-weighted 3D-MPRAGE sequences were as follows: TE = 1.64 ms; TR = 2,530 ms; TI = 900 ms; FOV = 256 mm × 256 mm; acquisition matrix size = 256 × 256 × 176; resolution = 1.0 × 1.0 × 1.0 mm^3^.

Demographic and clinical characteristics of Kyoto and COBRE datasets are provided in Supplementary Table 1. There was no significant difference between the two datasets with the exception of the sex ratio.

### Division of Train, Validation, and Test

The 3D-CAE was trained using the Kyoto dataset. The dataset was randomly partitioned into training data, validation data, and test data (138 subjects, 16 subjects, and 18 subjects, respectively). Training data, validation data, and test data were used for the training of the 3D-CAE, the validation of the model during training, and the final evaluation of generalizability within the datasets independent of the training and validation data, respectively. The COBRE dataset (142 subjects) was also used to evaluate the applicability of the network to another dataset.

The regression analysis was carried out using the COBRE dataset. The bias between MRI scanning sites might have affected the distribution of features extracted by 3D-CAE; thus, affecting the prediction error of the regression. Therefore, to avoid the scanning site effect, we used a single dataset for the regression. Then the fivefold cross-validation technique was applied. Namely, the COBRE dataset samples (142 subjects) were randomly divided into five subgroups (four groups for training and one group for validation) and cross-validated by changing the combinations of groups. This fivefold cross-validation process was repeated ten times. Note that only patients with schizophrenia had clinical information available for analysis, and regressions based on the clinical information were performed using data from patients with schizophrenia (71 subjects). The details for the division of data are shown in Table 1.

**TABLE 1:** Division of dataset.

		**Kyoto**	**COBRE**
**3D-CAE**			
(recon. error)	Train	✓	
	Validation	✓	
	Test	✓	✓
**Regression**			
(pred. error)	Train		✓
	Validation		✓

*The Kyoto dataset was used to develop the 3D-CAE model and was divided into train, validation and test dataset. The COBRE dataset was prepared for regression. At regression, fivefold cross-validation was performed.*

*3D-CAE, three-dimensional convolutional autoencoder; COBRE, Center for Biomedical Research Excellence.*

### MRI Preprocessing

The preprocessing was conducted using Statistical Parametric Mapping (SPM12, Wellcome Department of Cognitive Neurology, London, United Kingdom^3^) with the Diffeomorphic Anatomical Registration Exponentiated Lie Algebra (DARTEL) registration algorithm (Ashburner, 2007). All of the T1 whole-brain structural MRI scans were segmented into gray matter (GM), white matter, and cerebrospinal fluid. Individual GM images were normalized to the standard Montreal Neurological Institute (MNI) template with a 1.5 × 1.5 × 1.5 mm^3^ voxel size and modulated for GM volumes. All normalized GM images were smoothed with a Gaussian kernel of 8 mm full width at half maximum (FWHM). Subsequently, each image was cropped to remove the background as much as possible. The GM area was extracted from original images using a binary mask, created using SPM12. As a result, the size of input images to the 3D-CAE was 121 × 145 × 121 voxels.

Subsequently, the range of signal intensities in each image was normalized with a mean of 0 and a standard deviation of 1. The standardized value of voxel *i* in the sample *s*, x′s,i, was calculated as follows:


(4) x′s,i={xs,i-μsσs(i∈GM)   0   (o⁢t⁢h⁢e⁢r⁢w⁢i⁢s⁢e)

where *x*_*s, i*_ is the original value of intensity. μ_*s*_ and σ_*s*_ were average and standard deviation of all voxels contained in the GM area of sample *s*, respectively.

## Results

### Technical Evaluations: Reproduction Capability Performance

**FIGURE 3:**
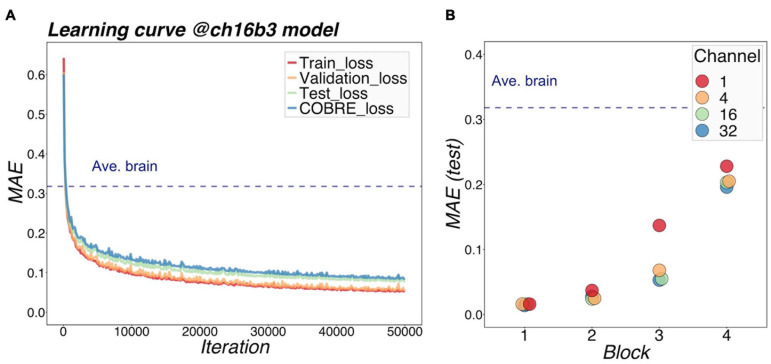
Learning performance of models. **(A)** shows the learning loss curve for a 16-channel and 3-block model. The red line shows the training loss, indicating that the learning has progressed, and the loss has fallen sufficiently. The validation loss and test loss were also decreased, so the model was not overfitting. The blue line indicates the loss at the other site (COBRE), and the loss degraded as well. It can be seen that the MAE of our proposed models was well below the level of Ave.brain at which the model was assumed to output the average brain. This suggested that our 3D-CAE models have successfully reproduced the brain images with individual characteristics. Similar learning curves were found for other models. In **(B)**, the reproduction performance of each of the 16 models were compared. The relationships between MAE, number of channels, and number of blocks are shown. The horizontal axis indicates the number of blocks, which is color-coded by the number of channels. As the number of blocks increased, the MAE tended to be larger, and as the number of channels increased, the MAE tended to be slightly smaller. 3D-CAE, three-dimensional convolutional autoencoder; COBRE, Center for Biomedical Research Excellence; MAE, mean absolute error.

Figure 3A shows a representative example of learning curves for the 3D-CAE with 16 channels and 3 blocks. Progressive decreases were shown not only with “train loss” (red line), but also “validation loss” (orange line) and “test loss” (green line); this indicated that the 3D-CAE successfully learned without overfitting. The level of MAEs were well below the level of the “average brain” (dashed line) (see section “Materials And Methods” for details). In addition, the curve for “COBRE loss” (blue line) showed a similar trend. This indicated that the 3D-CAE could be applied to MRI data from another site with different scanning parameters. Similar trends of learning curves were observed for the other fifteen 3D-CAEs with different hyperparameter settings.

**FIGURE 4:**
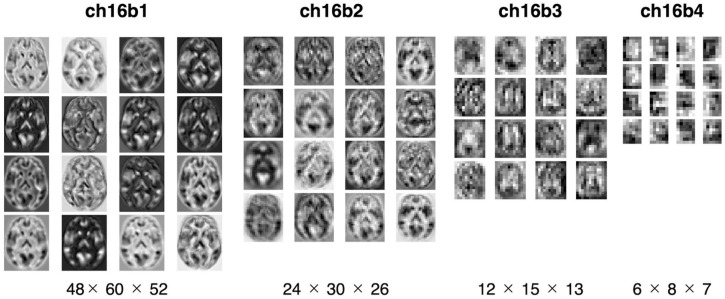
Visualization of extracted feature. The extracted features were mapped for four models with 16 channels. From left to right: the model with one, two, three, and four blocks. The middle slices of the horizontal slice from 3D feature are shown. In the one-block model, the morphology of the brain can be seen, but with four blocks, the images are more abstract.

Figure 3B summarized the reproduction performances (MAEs for the COBRE dataset) of the sixteen 3D-CAE models with respect to the number of channels and number of blocks. Regarding the number of blocks, it can be seen that the larger the number of blocks, the larger the reproduction error. This result is intuitively understandable, in that models with smaller blocks are easier to reconstruct because extracted latent features do not abstract the original image as much (Figure 4). Regarding the number of channels, although the differences were small, there was a tendency for the larger number of channels to be associated with smaller reproduction errors (see Supplementary Table 2 for more details). This result is consistent with the fact that the models with more channels have more expressive capability.

### Clinical Evaluation: Relation to Clinical Information

The efficacy of the proposed method was evaluated using linear regressions for predicting demographic and clinical information related to a psychiatric disorder, i.e., schizophrenia. Demographic and clinical information, including age, the dose of antipsychotic medication (CPZE), and scores of positive and negative symptoms (PANSS), were used as an objective variable, and all extracted features of 3D-CAE were used as explanatory variables. Feature using the ROI-based method was also used for comparison with the conventional method. A linear regression analysis was used as the simplest method to confirm if extracted features from 3D-CAEs with different hyperparameters (numbers of blocks and channels) preserved useful information. Each of the 16 3D-CAE models were analyzed 10 times, and the difference in predictive performance of the models was examined statistically.

**FIGURE 5:**
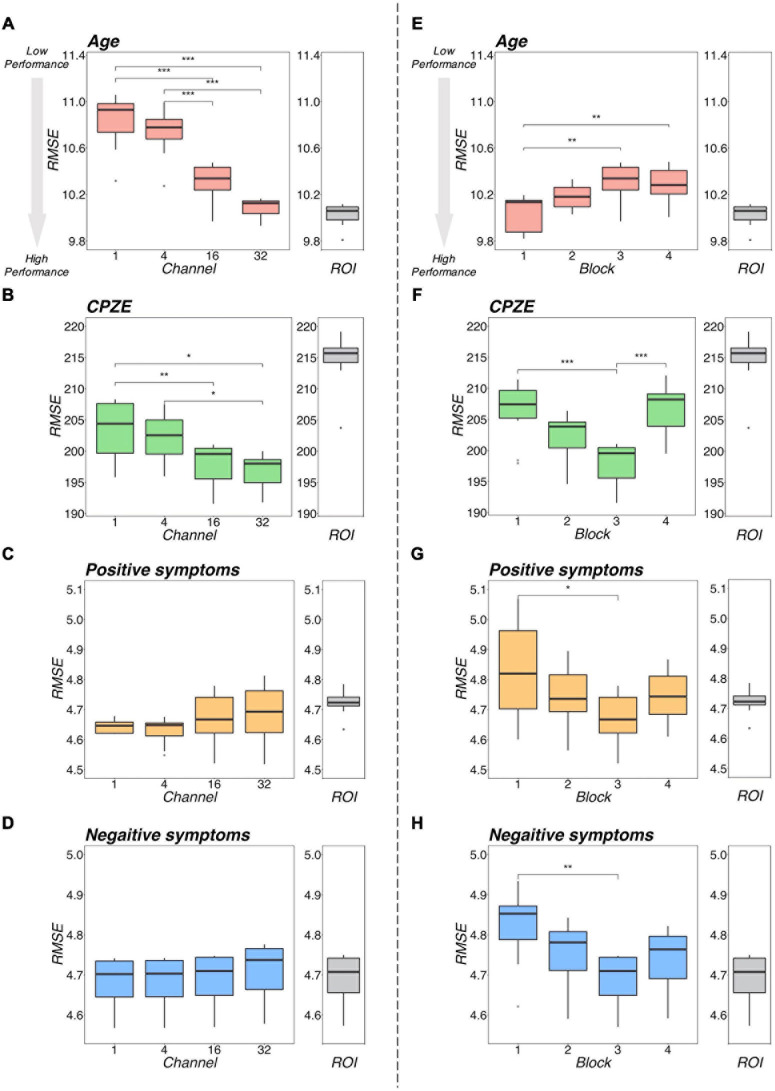
Regression performance plot. The left side **(A–D)** shows the model differences by number of channels for the four models with 3 blocks as an example. The right side **(E–H)** shows the model differences by number of blocks for the four models, with 16 channels as representative examples. Regarding age, as shown in **(A,E)**, the RMSEs were smaller with increasing number of channels and decreasing number of blocks. Regarding CPZE, as shown in **(B,F)**, the RMSEs were smaller with increasing number of channels. On the other hand, the RMSEs may be smaller in block 3. Regarding positive symptoms and negative symptoms, as shown in **(C,D)**, there was no apparent trend in the number of channels. As shown in **(G,H)**, the RMSE may be smaller in block 3. The results of each regression with the ROI method is also included for reference. It suggests that a model with 3 blocks may be appropriate for extracting schizophrenia-related information. ***p < 0.001, **p < 0.01, *p < 0.05 (two-way analysis of variance followed by Tukey’s multiple comparison test). CPZE, chlorpromazine equivalent; RMSE, root mean square error; ROI, region of interest.

Figure 5 illustrates a representative example of the regression analysis results. Differences in the performance of regression models (RMSE) with respect to the number of channels with 3 blocks (Figures 5A–D) and respect to the number of blocks with 16 channels (Figures 5E–H) were demonstrated as representative examples. The results of the comparison with the ROI method are shown in Table 2. The detailed results are described in Supplementary Tables 3–5, respectively.

**TABLE 2:**
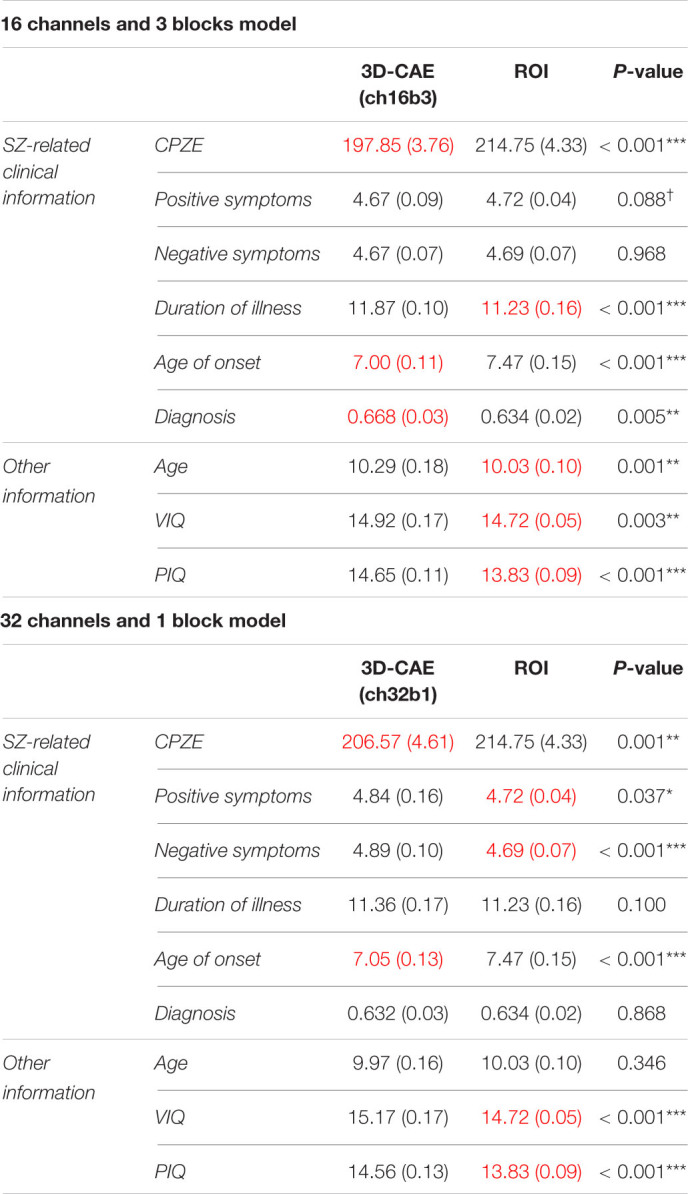
The results of the *t*-test.

The differences between 3D-CAE and ROI are presented as mean (standard deviation) and p-value of RMSE. Regarding the diagnosis, it is presented as accuracy. The significantly better performances are marked in red. The 3D-CAE model with 16 channels and 3 blocks was superior to the ROI method in predicting CPZE, age of onset, and diagnosis. The model also appeared comparable or better than the ROI method in positive symptoms. The 3D-CAE model with 32 channels and 1 block was also superior to the ROI method in predicting the CPZE and age of onset. Meanwhile, that the model was comparable to the ROI method for age prediction is different from the model with 16 channels and 3 blocks. ***p < 0.001, **p < 0.01, *p < 0.05, ^†^p < 0.1 (t-test).

*3D-CAE, three-dimensional convolutional autoencoder; ROI, region of interest; SZ, Schizophrenia; CPZE, chlorpromazine equivalent.*

Regarding the prediction of age, there were tendencies for the RMSEs to be smaller with increases in the number of channels (Figure 5A) and with decreasing number of blocks (Figure 5B). Indeed, statistical analysis revealed that there were significant differences between the models (channel: *p* < 0.001; block: *p* < 0.001). However, even the model with 32 channels and 1 block, which is considered one of the most predictive models, is equivalent to the ROI method (*p* = 0.346; Table 2), suggesting that for the prediction of age, 3D-CAE-based features were comparable to a conventional method.

In addition, the superiority of the 1 block condition was observed in the prediction of VIQ, PIQ and duration of illness (Supplementary Tables 2–4). However, 3D-CAEs with 1 block were not superior to the ROI method in predicting those information (VIQ: *p* < 0.001; PIQ: *p* < 0.001; duration of illness: *p* = 0.100; Table 2).

Regarding the prediction for CPZE, there was a tendency for the RMSEs to be smaller with increases in the number of channels (Figure 5C); on the other hand, the RMSEs were smallest with the condition of 3 blocks (Figure 5D). Statistical analysis revealed that there were significant differences between the models (channel: *p* < 0.001; block: *p* < 0.001). *Post hoc* analysis revealed that there were significant differences between 1 block and 3 blocks, and 3 blocks and 4 blocks. Moreover, the lowest level of RMSE of 3D-CAE was significantly lower than the RMSE from the ROI-based feature (*p* < 0.001; Table 2), indicating that for the prediction of CPZE, 3D-CAE based features outperformed a conventional method.

Regarding the prediction of positive symptoms, there was no clear tendency with respect to the number of channels (Figure 5E). On the other hand, with respect to the number of blocks, the RMSEs seemed to be the smallest with the condition of 3 blocks (Figure 5F). Statistical analysis indicated that there were significant differences between the models (channel: *p* < 0.001; block: *p* < 0.001). *Post hoc* analysis revealed that there were significant differences between 1 block and 3 blocks. Similar trends could be observed in the prediction of negative symptoms (Figures 5G,H), where there were significant differences between the models (channel: *p* < 0.001; block: *p* < 0.001). In comparison to the conventional method, the 3D-CAE model with 3 blocks showed a trend toward a smaller prediction error for positive symptoms than the ROI method (*p* = 0.088; Table 2), the mean RMSE (SD) was 4.67 (0.09) and 4.72 (0.04), respectively, suggesting that the 3D-CAE might be comparable or better than the ROI method. Regarding the prediction of negative symptoms, there was no significant difference between 3D-CAE and the conventional method (*p* = 0.968; Table 2).

In addition, the superiority of the 3 blocks condition was observed in the prediction of age of onset and diagnosis (Supplementary Tables 2–4). Furthermore, 3D-CAEs with 3 blocks performed better than the ROI method in predicting those clinical information (age of onset: *p* < 0.001; diagnosis: *p* = 0.005; Table 2).

To summarize the regression analysis results, in terms of clinical information related to schizophrenia, specifically for predicting CPZE, positive symptom score, age of onset, and diagnosis, 3D-CAE with 3 blocks had better prediction than other numbers of blocks models, regardless of the number of channels. In addition, 3D-CAE with 3 blocks performed better than the ROI method in predicting clinical information. On the other hand, in terms of information not directly related to schizophrenia, such as age and intelligence, 3D-CAE with 1 block had better prediction than 3D-CAE with other numbers of blocks, regardless of the number of channels. However, 3D-CAE with 1 block did not perform better than the ROI method in predicting information not directly related to schizophrenia.

**FIGURE 6:**
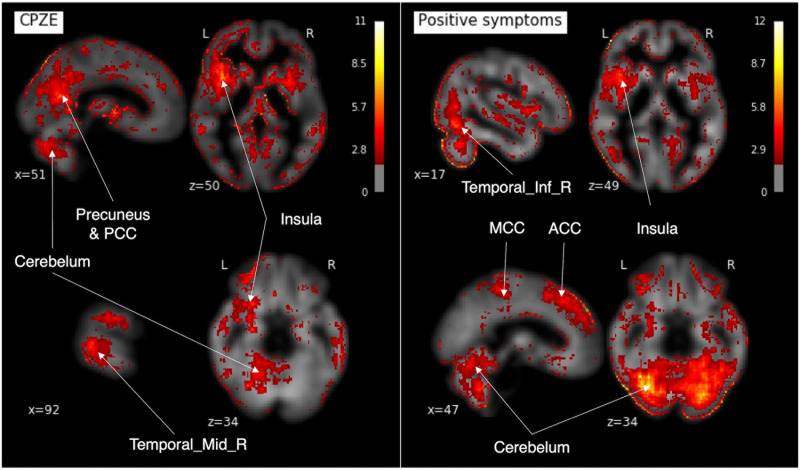
The saliency maps. In our developed 3D-CAE model, the saliency maps of the signals contributing to the prediction of each clinical information were obtained by calculating the gradient of the neural network. 3D-CAE, three-dimensional convolutional autoencoder; PCC, posterior cingulate cortex; MCC, middle cingulate cortex; ACC, anterior cingulate cortex; Temporal_Mid_R, right middle temporal gyrus; Temporal_Inf_R, right inferior temporal gyrus.

The saliency map was calculated to examine the correspondence between the features and the brain (Figure 6). The map showed that the regions contributing to CPZE prediction using 3D-CAE were the cerebellum, right middle temporal gyrus, the insula, posterior cingulate cortex, and precuneus. The regions that contributed to predicting the positive symptoms were found to the cerebellum, right inferior temporal gyrus, the insula, anterior and middle cingulate cortex. The other visualization results are described in Supplementary Figure 1.

## Discussion

We have shown that (1) the proposed 3D-CAEs successfully reproduced 3D MRI data with sufficiently low errors, and (2) the diagnostic label-free features extracted using 3D-CAE retained the relation of various clinical information. In addition, we explored the appropriate hyperparameter range of 3D-CAE, and our results suggest that a model with 3 blocks-based features might preserve information related to the medication dose and the severity of positive symptoms in patients with schizophrenia.

The reproduction errors of 3D-CAE were lower than the average brain level, indicating that the proposed 3D-CAEs successfully reproduced 3D brain MRI data with individual characteristics. In addition, the 3D-CAE trained with the Kyoto dataset was applicable to the COBRE dataset with different scanners and scanning parameters. Although the current study was tested using only two datasets, the results suggested that the proposed method may have applicability to data from multiple sites and scanners, itself a challenging issue in neuroimaging studies (Jovicich et al., 2009; Schnack et al., 2010; Fortin et al., 2018; Dewey et al., 2019; Yamashita et al., 2019).

Regression analyses demonstrated that the 3D-CAE-based feature was comparable or more effective than the ROI-based feature in predicting the medication dose and the severity of positive symptoms in patients with schizophrenia, even though 3D-CAE-based features were extracted without using a diagnostic label of schizophrenia. Because this approach enabled us to extract neuroimaging features of individuals without information on the clinical diagnosis, it can be useful for heterogeneous population data. Furthermore, using this approach, we were able to predict clinical variables. These imply that our approach in this study could be an alternative method to conventional methods based on categorical diagnostic information. This study showed that the prediction of CPZE, positive symptoms, and age of onset might be more improved in 3D-CAE than ROI. These are clinically meaningful because the model would help clinicians decide the treatment plan by predicting based on an objective indicator. In contrast, the current medication dose is mainly adjusted based on the patient’s self-reported condition.

Regarding the number of channels, 16− to 32-channel models demonstrated better performance. This is easy to understand because the more channels the model has, the more expressive it is (Zhu et al., 2019). However, since increasing number of channels inevitably results in increasing computational power needs, estimation of the appropriate number of channels is still important. Our results suggest that the number of channels may be sufficient at 16 or 32 for reproducing structural brain MRI scans. Regarding the number of blocks, our results indicated that information from a local receptive field (small number of block) was sufficient for predicting age. However, predicting schizophrenia-related clinical data required information from more global receptive fields (larger block numbers, such as 3-block). As the number of blocks increase, the effective receptive fields expand, and the global feature of the brain can be extracted (Szegedy et al., 2015; Le and Borji, 2017; Luo et al., 2017). In our model, the 3 blocks model contained eight convolutional layers, and effective receptive fields of the feature unit were about 68 × 68 × 68 voxels, corresponding to about 30% of the brain. This fact is consistent with the previous neuroimaging studies showing that the medication dose and symptoms severity are associated with the volume of multiple brain regions, including the temporal lobe, frontal lobe, and various subcortical regions (García-Martí et al., 2008; Palaniyappan et al., 2013; Van Erp et al., 2016; van Erp et al., 2018; Bullmore, 2019; Fan et al., 2019). The 3D-CAE-based feature’s superiority may be related to the detection of local signal interactions inherent in the convolutional methods; this contrasts with the ROI-based method, in which signals within each ROI are averaged and the interactions of local signals are discarded.

In our model, the saliency map showed that the cerebellum, temporal lobe, cingulate gyrus, and insular cortex had greater contributions in predicting the severity of symptoms and dose of antipsychotic medication. The present study results were consistent with the results of previous studies showing that positive symptoms and CPZE correlated with cortical thickness thinning in the temporal lobe (van Erp et al., 2018), and that cerebellar atrophy was associated with positive symptoms (Cierpka et al., 2017). The insular and cingulate cortices, which were shown to be significant contributors to clinical variables in the present study, have been repeatedly reported to be reduced in the regional brain volume in schizophrenia (Glahn et al., 2008; Takayanagi et al., 2013; Gupta et al., 2015; Uwatoko et al., 2015). However, the relationship between these areas and positive symptoms and CPZE requires further investigation. As a side note, because the relatively high values of the edge of the brain may be influenced by the traits of Smoothgrad (Smilkov et al., 2017) that emphasize the edge, it was difficult to consider them from a neuroimaging study perspective.

There are some limitations to our study. First, this study only explored a limited range of hyperparameters. In CAE, there are several hyperparameters than those explored, such as activate function, optimizer, and learning rate. However, because we focused on the total dimension of the extracted features and the effective receptive field’s size, the numbers of blocks and channels were explored as the target variables. In addition, the exploration range of hyperparameters was limited due to practical reasons including the computational power and costs of the experiments.

Second, the differences in preprocessing of neuroimaging data may affect the robustness of the study results. In this study, we employed the standard preprocessing methods (e.g., image resolution, standardization, smoothing), which have been used in neuroimaging studies, such as voxel-based morphometry (Ashburner and Friston, 2000). Nevertheless, further studies may evaluate the effects of the preprocessing methods on results.

Third, the datasets used in this study only included patients diagnosed with schizophrenia as well as healthy subjects. Considering the heterogeneity of psychiatric disorders, it will be necessary to examine the applicability of diagnostic label-free feature extraction using 3D-CAE to other psychiatric disorders in the future.

Fourth, regressions were used to predict clinical and demographic scores, but the 3D-CAE-based feature outperformed the feature of the ROI does not necessarily prove that the predictive value generated is clinically useful. In the present study, the main goal was feature extraction, and only simple regression was used for prediction. The additional experiments with the development of a fine-tuned model and evaluation using longitudinal data of disease process are needed in the future. These may improve clinical decisions for assessing patients’ prognosis and estimating an appropriate medication dose.

In this paper, we presented 3D-CAE-based feature extraction for brain structural imaging of psychiatric disorders. We found that 3D-CAE can extract features that retained their relation to clinical information from 3D MRI data without diagnostic labels. Our data suggest that 3D-CAE models with effective hyperparameter settings may extract information related to the medication dose and the severity of symptoms in patients with schizophrenia. The feature extraction without using diagnostic labels based on the current diagnostic criteria is scientifically significant and may lead to the development of alternative data-driven diagnostic criteria.

## Data Availability Statement

All data generated or analyzed during this study are included in this published article. The primary data can be obtained from public databases, including the Decoded Neurofeedback (DecNef) Project Brain Data Repository (https://bicr-resource.atr.jp/srpbs1600/) and the Centers for Biomedical Research Excellence (COBRE; http://fcon_1000.projects.nitrc.org/indi/retro/cobre.html).

## Ethics Statement

All study participants signed an informed consent form. The study was performed in accordance with the current Ethical Guidelines for Medical and Health Research Involving Human Subjects in Japan and was approved by the Committee on Medical Ethics of Kyoto University and National Center of Neurology and Psychiatry.

## Author Contributions

HY, YH, and YY conceived, designed the research, and drafted the manuscript. HY and YH conducted the deep learning experiments and analyzed the data. GS, JM, TM, and HT collected MRI data. GS, JM, TM, HT, MH, and AH provided critical revisions. All authors contributed to and have approved the final manuscript.

## Conflict of Interest

The authors declare that the research was conducted in the absence of any commercial or financial relationships that could be construed as a potential conflict of interest.

## Footnotes

1
http://fcon_1000.projects.nitrc.org/indi/retro/cobre.html

2
https://www.python.org/

3
https://www.fil.ion.ucl.ac.uk/spm/software/spm12/
