# Supplementary material for: Three-Dimensional Convolutional Autoencoder Extracts Features of Structural Brain Images With a “Diagnostic Label-Free” Approach: Application to Schizophrenia Datasets
Source: Front Neurosci. 2021 Jul 7;15:652987. doi: 10.3389/fnins.2021.652987 (PMC8294943; doi:10.3389/fnins.2021.652987)
Supplement: Supplementary file 1 [file Data_Sheet_1.PDF]

Supplementary Material

1. Supplementary Figure

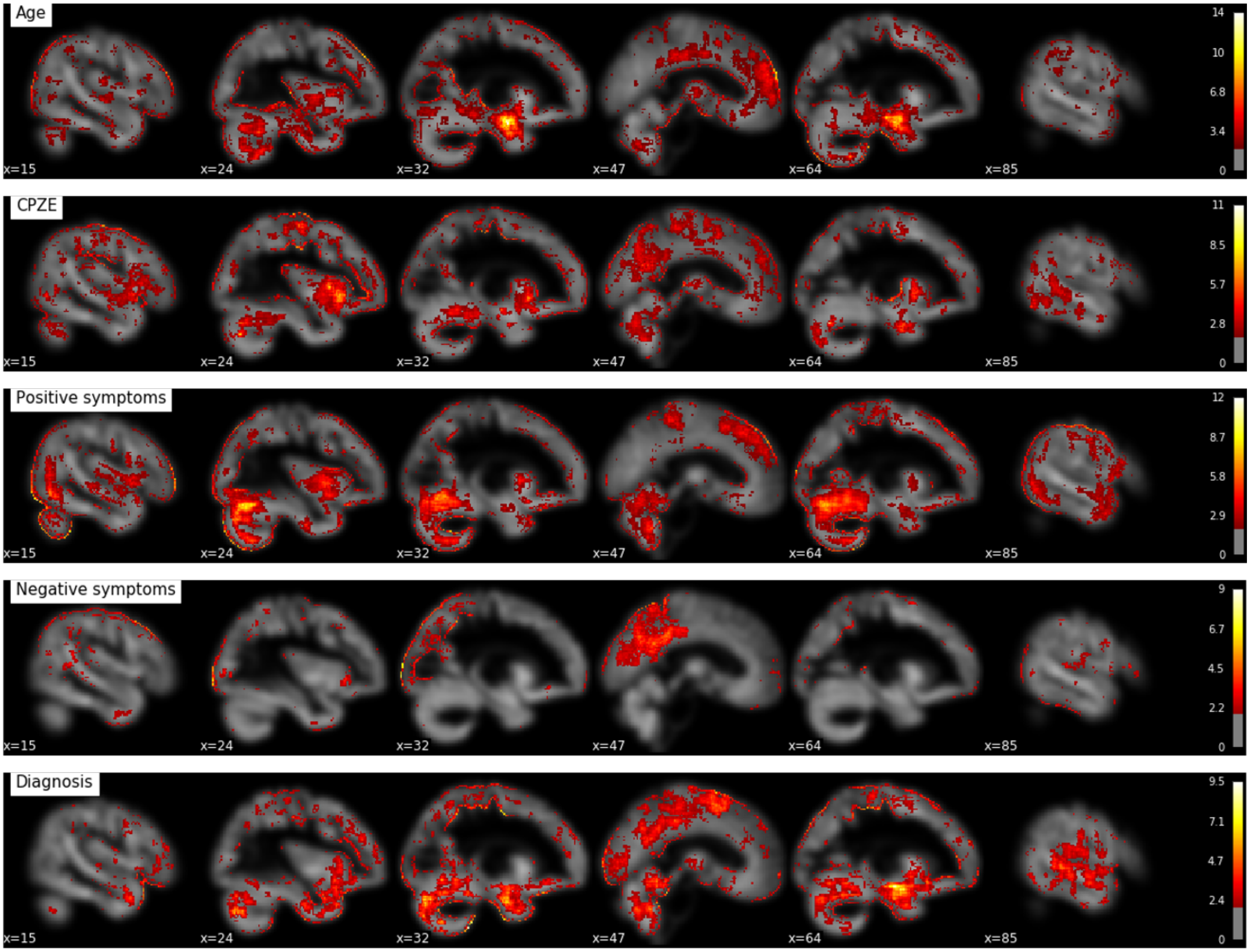

Supplementary Figure 1. The saliency maps for each of demographic and clinical information. In our developed CAE model, the saliency maps of the signals contributing to the prediction of each demographic and clinical information was obtained by the gradient of the neural network.

2. Supplementary Tables

	Kyoto (n=172)	COBRE (n=142)	P value
Healthy / SZ	87 / 85	71 / 71	1
Male / Female	96 / 76	108 / 34	< 0.001
Age	35.6 ± 9.4	36.9 ± 12.9	0.29
VIQ	-	100.1± 6.9	-
PIQ	-	102.2± 15.0	-
CPZE	555.6 ± 372.5	407.6 ± 1072.6	0.24
Positive symptoms	14.0 ± 4.5	15.0 ± 4.9	0.18
Negative symptoms	15.3 ± 5.3	14.6 ± 4.8	0.40
Duration of illness	13.3 ± 8.5	15.6 ± 12.6	0.17
Age of onset	-	22.2 ± 8.5	-

Supplementary Table 1. Demographic and clinical characteristics of the datasets. Where values are shown as number or mean ± standard deviation. P values refer to t-test or chi-square test.

	Train loss	Valid loss	Test loss	COBRE loss
Ave. Brain	0.318	-	-	0.333
ch1b1	0.016	0.016	0.016	0.023
ch4b1	0.016	0.016	0.016	0.023
ch16b1	0.014	0.014	0.016	0.020
ch32b1	0.015	0.015	0.014	0.018
ch1b2	0.037	0.038	0.037	0.050
ch4b2	0.026	0.027	0.025	0.040
ch16b2	0.026	0.025	0.024	0.032
ch32b2	0.024	0.024	0.028	0.034
ch1b3	0.114	0.131	0.137	0.190
ch4b3	0.065	0.071	0.068	0.102
ch16b3	0.053	0.057	0.055	0.080
ch32b3	0.050	0.054	0.053	0.079
ch1b4	0.179	0.337	0.228	0.33
ch4b4	0.161	0.212	0.205	0.301
ch16b4	0.155	0.211	0.203	0.293
ch32b4	0.149	0.203	0.196	0.286

Supplementary Table 2. Reconstruction error for each model. Where the 'Ave. Brain' of train loss was the average brain in the Kyoto dataset and the 'Ave. Brain' of COBRE loss was the average brain in the COBRE dataset.

	channel	block	interaction
Age	< 0.001	< 0.001	< 0.001
Positive symptoms	< 0.001	< 0.001	< 0.001
Negative symptoms	< 0.001	< 0.001	0.047
CPZE	< 0.001	< 0.001	0.6126
VIQ	0.3186	< 0.001	< 0.001
PIQ	< 0.001	0.025	0.112
Duration of illness	< 0.001	< 0.001	< 0.001
Age of onset	0.002	< 0.001	0.017
Diagnosis	< 0.001	0.506	< 0.001

Supplementary Table 3. The results of ANOVA. Since there are significant differences in most demographic and clinical information, it can be said that there were differences in the performances depending on the hyper parameters.

	Age	CPZE	Positive symptoms	Negative symptoms	VIQ	PIQ	Duration of illness	Age of onset	Diagnosis
ROI	10.03 (0.01)	214.75 (4.33)	4.72 (0.04)	4.69 (0.07)	14.72 (0.05)	13.83 (0.09)	11.23 (0.16)	7.47 (0.15)	0.63 (0.02)
ch1b1	10.08 (0.16)	208.79 (4.54)	4.67 (0.03)	4.69 (0.07)	14.92 (0.08)	14.59 (0.12)	11.43 (0.12)	7.04 (0.12)	0.66 (0.02)
ch4b1	10.21 (0.22)	206.94 (4.48)	4.66 (0.04)	4.70 (0.07)	14.91 (0.06)	14.69 (0.15)	11.46 (0.13)	7.10 (0.10)	0.64 (0.02)
ch16b1	10.04 (0.16)	206.40 (4.82)	4.83 (0.16)	4.82 (0.09)	14.98 (0.12)	14.56 (0.11)	11.43 (0.16)	7.08 (0.12)	0.64 (0.02)
ch32b1	9.97 (0.16)	206.57 (4.61)	4.84 (0.16)	4.89 (0.10)	15.17 (0.17)	14.56 (0.13)	11.35 (0.17)	7.05 (0.13)	0.63 (0.01)
ch1b2	10.38 (0.17)	208.14 (4.28)	4.66 (0.04)	4.68 (0.07)	14.87 (0.07)	14.67 (0.13)	11.95 (0.13)	7.02 (0.17)	0.65 (0.02)
ch4b2	10.42 (0.14)	203.72 (4.21)	4.64 (0.05)	4.69 (0.07)	14.92 (0.04)	14.61 (0.10)	11.71 (0.15)	7.01 (0.12)	0.67 (0.02)
ch16b2	10.18 (0.10)	201.98 (4.12)	4.75 (0.10)	4.75 (0.08)	14.88 (0.10)	14.55 (0.09)	11.60 (0.14)	6.96 (0.10)	0.64 (0.02)
ch32b2	10.06 (0.10)	201.01 (4.12)	4.77 (0.12)	4.79 (0.09)	14.86 (0.11)	14.52 (0.09)	11.59 (0.14)	6.89 (0.12)	0.66 (0.01)
ch1b3	10.83 (0.23)	203.50 (4.60)	4.64 (0.02)	4.68 (0.07)	14.83 (0.08)	14.73 (0.12)	11.94 (0.13)	7.17 (0.16)	0.66 (0.02)
ch4b3	10.73 (0.21)	202.33 (4.07)	4.63 (0.04)	4.68 (0.07)	14.84 (0.11)	14.70 (0.11)	12.03 (0.10)	6.95 (0.10)	0.66 (0.03)
ch16b3	10.29 (0.18)	197.85 (3.76)	4.67 (0.09)	4.69 (0.07)	14.91 (0.17)	14.65 (0.10)	11.87 (0.10)	7.00 (0.11)	0.67 (0.03)
ch32b3	10.09 (0.08)	196.85 (3.08)	4.69 (0.10)	4.71 (0.07)	14.80 (0.12)	14.53 (0.09)	11.78 (0.10)	6.96 (0.11)	0.68 (0.02)
ch1b4	10.47 (0.14)	209.73 (7.02)	4.65 (0.04)	4.68 (0.07)	14.88 (0.07)	14.75 (0.09)	11.83 (0.14)	7.19 (0.21)	0.69 (0.03)
ch4b4	10.72 (0.17)	207.91 (3.90)	4.71 (0.08)	4.71 (0.07)	14.86 (0.13)	14.69 (0.12)	11.95 (0.13)	7.31 (0.27)	0.66 (0.02)
ch16b4	10.28 (0.15)	207.04 (4.09)	4.75 (0.09)	4.74 (0.08)	14.86 (0.12)	14.55 (0.12)	11.92 (0.13)	7.16 (0.16)	0.70 (0.03)
ch32b4	10.07 (0.13)	205.11 (4.55)	4.75 (0.15)	4.79 (0.09)	14.83 (0.15)	14.53 (0.10)	11.67 (0.12)	7.06 (0.08)	0.67 (0.02)

Supplementary Table 4. Regression performance for each model and ROI methods. Listed on Age, CPZE, Positive symptoms, Negative symptoms, VIQ, PIQ, Duration of illness, Age of onset and Diagnosis. The average of the regression results was shown. Red ink indicated better performance than that

of the ROI method. It seems that the CAE method was superior to the ROI method in predicting CPZE, Positive symptoms, Negative symptoms, Age of onset and Diagnosis.

Models with 1 block

	ch4-ch1	ch16-ch1	ch32-ch1	ch16-ch4	ch32-ch4	ch32-ch16
Age	0.387	0.939	0.470	0.147	0.020	0.811
CPZE	0.806	0.656	0.705	0.994	0.998	1.000
Positive symptoms	1.000	0.015	0.008	0.012	0.006	0.996
Negative symptoms	0.986	0.007	< 0.001	0.018	< 0.001	0.354
VIQ	0.997	0.678	< 0.001	0.553	< 0.001	0.003
PIQ	0.321	0.957	0.960	0.132	0.134	1.000
Duration of illness	0.969	1.000	0.688	0.983	0.415	0.636
Age of onset	0.666	0.810	0.992	0.994	0.822	0.927
Diagnosis	0.400	0.174	0.092	0.956	0.840	0.989

Models with 2 blocks

	ch4-ch1	ch16-ch1	ch32-ch1	ch16-ch4	ch32-ch4	ch32-ch16
Age	0.911	0.007	< 0.001	0.001	< 0.001	0.197
CPZE	0.103	0.011	0.003	0.788	0.477	0.954
Positive symptoms	0.954	0.108	0.031	0.034	0.008	0.944
Negative symptoms	0.999	0.157	0.018	0.203	0.026	0.775
VIQ	0.478	0.971	1.000	0.747	0.430	0.953
PIQ	0.587	0.066	0.015	0.568	0.235	0.923
Duration of illness	0.002	< 0.001	< 0.001	0.360	0.290	0.999
Age of onset	0.995	0.743	0.135	0.864	0.210	0.621
Diagnosis	0.094	0.239	0.565	0.001	0.693	0.014

Models with 3 blocks

	ch4-ch1	ch16-ch1	ch32-ch1	ch16-ch4	ch32-ch4	ch32-ch16
Age	0.664	< 0.001	< 0.001	< 0.001	< 0.001	0.076
CPZE	0.909	0.013	0.003	0.067	0.017	0.941

Positive symptoms	0.952	0.838	0.484	0.532	0.221	0.930
Negative symptoms	1.000	0.996	0.780	0.998	0.797	0.883
VIQ	1.000	0.491	0.948	0.534	0.927	0.218
PIQ	0.921	0.312	0.001	0.680	0.006	0.092
Duration of illness	0.229	0.484	0.014	0.009	< 0.001	0.301
Age of onset	0.001	0.015	0.002	0.809	0.999	0.875
Diagnosis	0.974	0.983	0.715	0.860	0.457	0.897

Models with 4 blocks

	**ch4-ch1**	**ch16-ch1**	**ch32-ch1**	**ch16-ch4**	**ch32-ch4**	**ch32-ch16**
Age	0.004	0.031	< 0.001	< 0.001	< 0.001	0.016
CPZE	0.851	0.635	0.190	0.980	0.605	0.827
Positive symptoms	0.433	0.131	0.097	0.889	0.822	0.999
Negative symptoms	0.835	0.361	0.015	0.847	0.106	0.429
VIQ	0.984	0.954	0.758	0.999	0.923	0.966
PIQ	0.616	0.001	< 0.001	0.023	0.012	0.995
Duration of illness	0.179	0.421	0.056	0.950	< 0.001	0.001
Age of onset	0.549	0.985	0.436	0.348	0.034	0.648
Diagnosis	0.001	0.377	0.078	< 0.001	0.366	0.001

Models with 1 channel

	b2-b1	b3-b1	b4-b1	b3-b2	b4-b2	b4-b3
Age	0.004	< 0.001	< 0.001	< 0.001	0.629	< 0.001
CPZE	0.992	0.126	0.978	0.213	0.904	0.053
Positive symptoms	0.987	0.536	0.677	0.739	0.858	0.996
Negative symptoms	0.984	0.981	0.981	1.000	1.000	1.000
VIQ	0.456	0.083	0.741	0.762	0.965	0.478
PIQ	0.407	0.038	0.013	0.604	0.355	0.974

Duration of illness	< 0.001	< 0.001	< 0.001	0.996	0.177	0.263
Age of onset	0.999	0.303	0.167	0.237	0.125	0.986
Diagnosis	0.691	0.818	0.005	0.216	< 0.001	0.044

Models with 4 channels

	**b2-b1**	**b3-b1**	**b4-b1**	**b3-b2**	**b4-b2**	**b4-b3**
Age	0.081	< 0.001	< 0.001	0.003	0.004	0.998
CPZE	0.326	0.082	0.954	0.878	0.131	0.025
Positive symptoms	0.852	0.538	0.146	0.947	0.024	0.006
Negative symptoms	0.930	0.880	0.999	0.999	0.869	0.805
VIQ	0.986	0.328	0.703	0.184	0.492	0.918
PIQ	0.489	0.993	0.999	0.335	0.406	0.999
Duration of illness	0.001	< 0.001	< 0.001	< 0.001	0.001	0.510
Age of onset	0.651	0.198	0.029	0.827	0.001	< 0.001
Diagnosis	0.003	0.123	0.221	0.427	0.267	0.989

Models with 16 channels

	**b2-b1**	**b3-b1**	**b4-b1**	**b3-b2**	**b4-b2**	**b4-b3**
Age	0.182	0.003	0.005	0.343	0.450	0.997
CPZE	0.106	< 0.001	0.986	0.144	0.051	< 0.001
Positive symptoms	0.344	0.013	0.329	0.414	1.000	0.432
Negative symptoms	0.242	0.003	0.105	0.258	0.971	0.490
VIQ	0.413	0.720	0.190	0.957	0.961	0.753
PIQ	0.998	0.235	0.998	0.167	1.000	0.168
Duration of illness	0.038	< 0.001	< 0.001	< 0.001	< 0.001	0.834
Age of onset	0.181	0.433	0.485	0.947	0.006	0.026
Diagnosis	0.924	0.031	< 0.001	0.006	< 0.001	0.049

Models with 32 channels

	b2-b1	b3-b1	b4-b1	b3-b2	b4-b2	b4-b3
Age	0.321	0.149	0.276	0.970	1.000	0.985
CPZE	0.024	< 0.001	0.858	0.130	0.139	< 0.001
Positive symptoms	0.602	0.066	0.444	0.554	0.994	0.714
Negative symptoms	0.075	< 0.001	0.100	0.224	0.999	0.177
VIQ	< 0.001	< 0.001	< 0.001	0.761	0.945	0.973
PIQ	0.840	0.958	0.968	0.988	0.982	1.000
Duration of illness	0.002	< 0.001	< 0.001	0.019	0.565	0.292
Age of onset	0.018	0.246	0.995	0.615	0.009	0.160
Diagnosis	0.053	0.002	0.009	0.532	0.891	0.916

Supplementary Table 5. The results of post-hoc analysis. The differences between the different hyper parameter for a model with a particular number of blocks or channels were shown. The numbers listed were p-values and red ink indicated a significant difference.
